# Genotyping-by-sequencing of *Brassica oleracea* vegetables reveals unique phylogenetic patterns, population structure and domestication footprints

**DOI:** 10.1038/s41438-018-0040-3

**Published:** 2018-07-01

**Authors:** Zachary Stansell, Katie Hyma, Jonathan Fresnedo-Ramírez, Qi Sun, Sharon Mitchell, Thomas Björkman, Jian Hua

**Affiliations:** 1 School of Integrative Plant Science, Horticulture Section, Cornell University, Geneva, NY 14456 USA; 2 Genomic Diversity Facility, Institute of Biotechnology, Cornell University, Ithaca, NY 14853 USA; 3 Bioinformatics Facility, Institute of Biotechnology, Cornell University, Ithaca, NY 14853 USA; 4 School of Integrative Plant Science, Plant Biology Section, Cornell University, Ithaca, NY 14853 USA; 5 Present Address: Syracuse University, Syracuse, NY USA; 6 Present Address: Department of Horticulture and Crop Science, The Ohio State University/OARDC, Wooster, OH 44691 USA

## Abstract

*Brassica oleracea* forms a diverse and economically significant crop group. Improvement efforts are often hindered by limited knowledge of diversity contained within available germplasm. Here, we employ genotyping-by-sequencing to investigate a diverse panel of 85 landrace and improved *B. oleracea* broccoli, cauliflower, and Chinese kale entries. Ultimately, 21,680 high-quality SNPs were used to reveal a complex and admixed population structure and clarify phylogenetic relationships among *B. oleracea* groups. Each broccoli landrace contained, on average, 8.4 times as many unique alleles as an improved broccoli and landraces collectively represented 81% of all broccoli-specific alleles. Commercial broccoli hybrids were largely represented by a single subpopulation identified within a complex population structure. Greater allelic diversity in landrace broccoli and 96.1% of SNPs differentiating improved cauliflower from landrace cauliflower were common to the larger pool of broccoli germplasm, supporting a parallel or later development of cauliflower due to introgression events from broccoli. Chinese kale was readily distinguished by principal coordinate analysis. Genotyping was accomplished with and without reliance upon a reference genome producing 141,317 and 20,815 filtered SNPs, respectively, supporting robust SNP discovery methods in neglected or unimproved crop groups that lack a reference genome. This work clarifies the population structure, phylogeny, and domestication footprints of landrace and improved *B. oleracea* broccoli using many genotyping-by-sequencing markers. Additionally, a large pool of genetic diversity contained in broccoli landraces is described which may enhance future breeding efforts.

## Which came first: the broccoli or the cauliflower?

A comparison of gene variations in modern-day offspring of wild cabbage shows that broccoli may have preceded the appearance of cauliflower in domesticated crops. Jian Hua, Thomas Bjorkman, and colleagues from Cornell University in the US examined the DNA sequences of 85 traditional “landrace” and improved broccoli, cauliflower, and Chinese kale plants, all of which descend from the species *Brassica oleracea*, or wild cabbage. They found 21,680 sequence variations among them all, which they used to look for patterns of relatedness between the plants. Their results support the possible domestication of cauliflower after or in parallel with broccoli. The study identifies a large pool of genetic diversity within broccoli landraces that could be used to improve future breeding efforts.

## Introduction

*Brassica oleracea* is an economically important and outcrossing species domesticated as early as 2000 BCE and has been specialized into many unique botanical types such as broccoli, cauliflower, cabbage, kale, Chinese kale, and Brussels sprouts. Commercial production of these crops is frequently subject to abiotic stressors such as heat stress typically resulting in a reduction in horticultural quality. Improvement efforts for these crop groups are often limited by a lack of knowledge of available diversity or genetic bottlenecks that occurred during domestication or dispersal. Early *B. oleracea* vegetables were grown in close geographical proximity with several sexually compatible undomesticated relatives^1^ and are grouped into a larger coenospecies (2n = 18) capable of sharing genetic information and producing fertile offspring^2^. While a consensus theory of *B. oleracea* domestication has not been reached, it is generally assumed that early domestication of *B. oleracea* occurred in the Mediterranean and was ultimately spread by trade resulting in a proliferation of locally adapted landraces representing a complex genetic admixture^3–5^. It is currently unclear if broccoli and cauliflower were domesticated independently, if one was selected from within the other, or the degree to which mutual introgression has occurred^6–8^. While early work indicated a wider genetic basis for cauliflower implying that broccoli was selected from cauliflower, an opposite broccoli-first domestication model has been suggested^7^. Tonguç and Griffiths (2004)^9^ demonstrated that diversity was lower in cauliflower than broccoli. Studies of molecular^4,10^ and morphological^5^ markers indicate a closer relationship between undomesticated *B. oleracea* and broccoli than cauliflower and suggest cauliflower may have experienced introgression from broccoli.

These studies have all relied on morphological observations or a small number of molecular markers (<200) that can lead to biased estimates based on analysis of limited genomic regions and weak inferences of population structure. Genotyping-by-Sequencing (GBS) is a technically straightforward, multiplexed approach that is highly suitable for population diversity studies by sequencing genomic subsets specifically targeted by restriction enzymes for fast, specific, and reproducible results^11^. The large numbers of single-nucleotide polymorphism (SNP) markers generated by GBS provide a deeper understanding of population structure and genetic diversity, are suitable for genome-wide association studies (GWAS) and even candidate gene discovery. Typically, GBS studies have relied on a high-quality reference genome to align sequencing reads; however, a quality reference genome may be unavailable or difficult to assemble in certain minor or neglected crops. Therefore we are also interested in quantifying SNP production without the benefit of a reference genome.

The main objectives of this study were (1) to investigate the diversity, population structure, and possible selection footprints of broccoli; (2) to address competing broccoli-first or cauliflower-first domestication models; and (3) to provide a comparison of SNP production without a high-quality reference genome for subsequent analyses of other neglected crop groups.

## Results

### Phylogenetic relationships

**Fig. 1:**
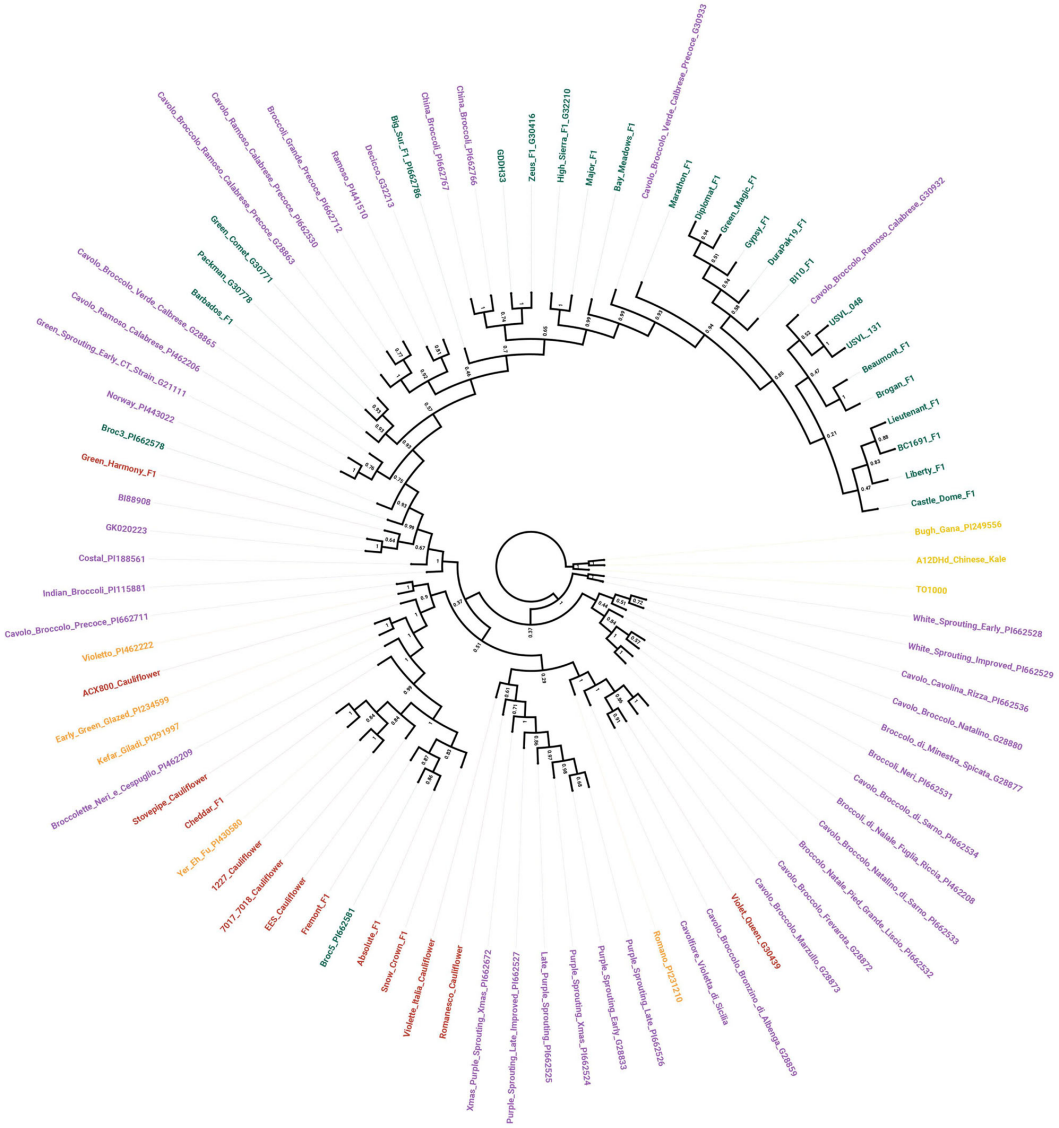
Maximum likelihood cladogram generated from all entries using 21,680 markers using phylogeny reconstruction with 2000 bootstrap replications . Botanical group is denoted by color: Chinese kale (yellow), landrace broccoli (purple), improved broccoli (green), landrace cauliflower (orange), improved cauliflower (red)

Phylogeny reconstruction using 21,680 markers revealed several interesting patterns of relatedness among entries (Fig. 1). Chinese kale entries formed a distinct clade and were thus chosen as an outgroup. Broccoli landraces with “purple sprouting” and “white sprouting” passport terms formed distinct clades. Recently released improved broccoli clustered relatively closely and entries from various breeding programs tended to locate into subclades. For example, the broccoli hybrids “Lieutenant F1” (2011), “Castle Dome F1” (2006), “Liberty F1” (1994), and “BC1691 F1” (2011) from Seminis/Peto; “Beaumont F1” (2003) and “Brogan F1” (1997) from Bejo; and “Diplomat F1” (2004), “Green Magic F1” (2004), and “Gypsy F1” (2002) from Sakata Seed Co.; “USVL 048” (2012) and “USVL 131” (2012) breeding lines from USDA-USVL all formed distinct subclades. Interestingly, two broccoli landraces, “Cavolo Broccolo Ramoso Calabrese” and “Cavolo Broccolo Verde Calbrese Precoce”, collocated within the clade otherwise comprised of improved broccoli. A clade of older broccoli hybrids: “Barbados F1” (1991), “Packman F1” (1983), and “Green Comet G30413” (1968), was isolated from most of the other improved broccoli entries. Excluding the putative broccoli–cauliflower hybrid “Green Harmony F1”, all improved and landrace cauliflower entries formed a clade which included the subclade of broccoli marked with “purple sprouting” passport terms as well as four other landrace broccoli entries “Cavolo Broccolo Frevarota”, “Cavolo Broccolo Marzullo, “Cavolfiore Violetta di Sicilia”, and “Broccolette Neri e Cespuglio”.

### Population structure

**Fig. 2:**
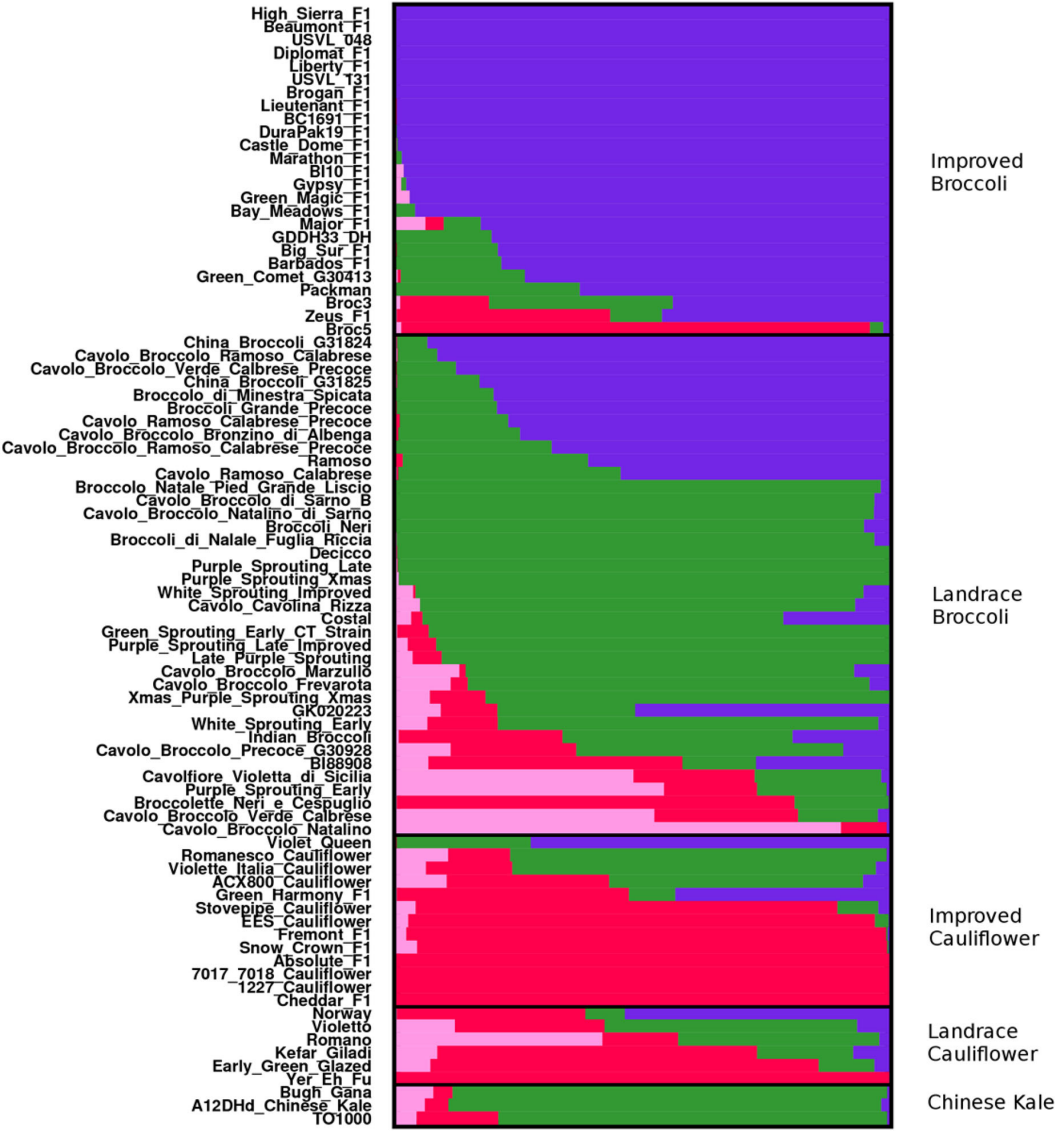
Population structure (K = 4) of all entries using 21,680 SNPs . Proportionate membership coefficients are represented by width of horizontal bar of a color

The population structure of entries included in this study was calculated with 21,680 unlinked GBS markers grouped into (K = 4) populations. In general, improved broccoli entries were largely associated (85.5%) with a single theoretical population denoted in purple (Fig. 2). F1 hybrid broccoli released since 2000 contained 94.3% membership in this purple group. Older improved broccoli hybrids, “Green Comet G30413” (1968), “Packman F1” (1983), and “Barbados F1” (1991), contained some limited membership within the green group (25.2%, 37.2%, 21.3%). Interestingly, aside from the putative broccoli–cauliflower hybrid “Green Harmony F1” and “Violet Queen”, improved cauliflower had little representation within the purple group (1.1%) but displayed some membership in the green group (21.3%) prevalent within landrace broccoli. Both landrace broccoli and landrace cauliflower were overall far more admixed than improved broccoli and improved cauliflower. Chinese kale entries were predominately represented by the green group (average = 85.0%).

**Fig. 3:**
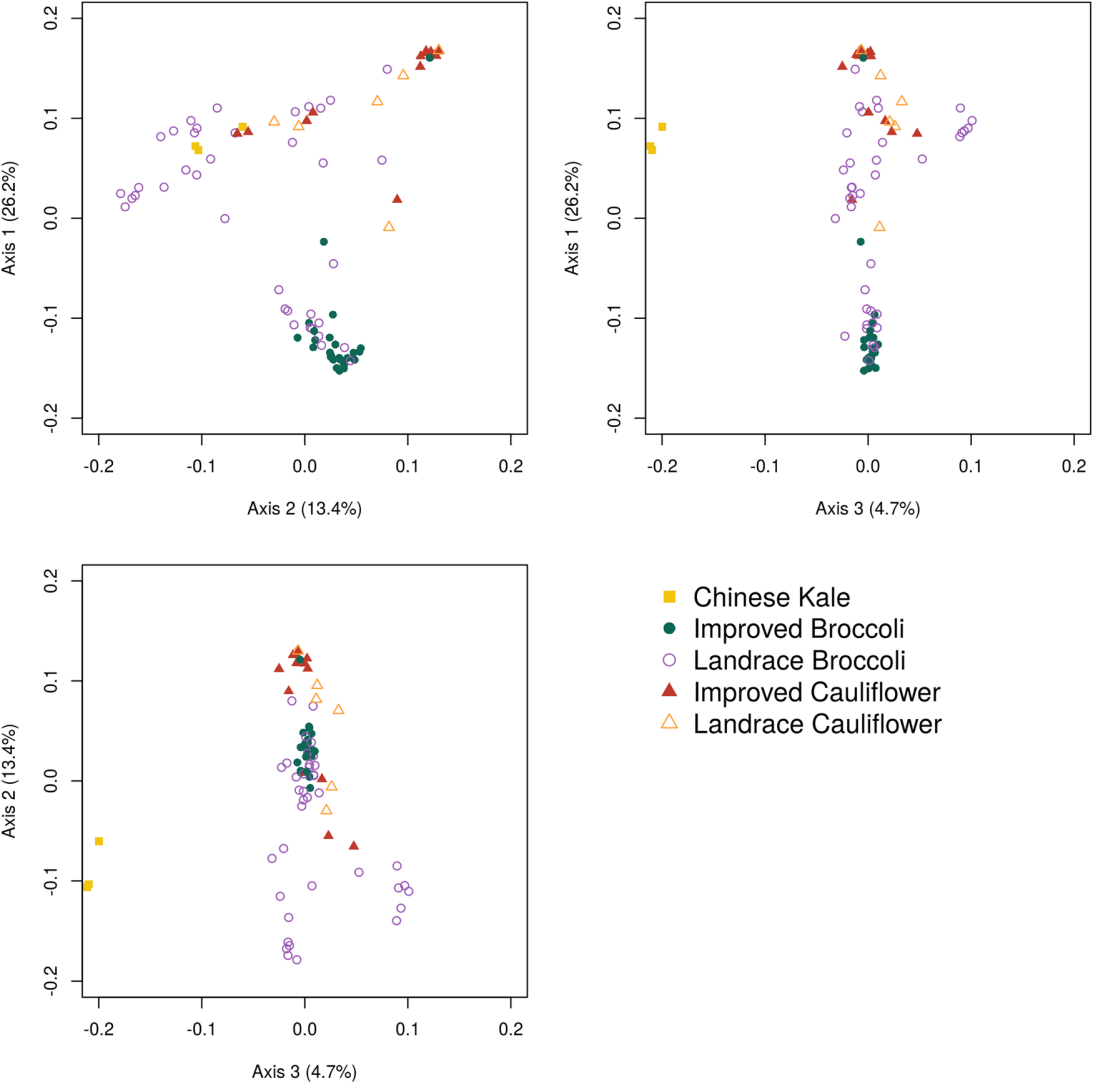
Principal coordinate analysis visualized by pairwise plotting of axes explaining the greatest variation among entries . Chinese kale is represented by squares, broccoli as circles, and cauliflower as triangles. Color scheme is as given in Fig. 1

Principal coordinate analysis (Fig. 3; Supplemental Figure SF1; Supplemental Movie SM1) was conducted using three axes explaining the greatest degree of variation (26.3%, 13.4%, 4.7%) among all entries. In comparisons between Axis 1 and Axis 2, a triangular shape is observed with improved cauliflower at one vertex, improved broccoli at another, and an admixed group of landrace broccoli and cauliflower forming the third vertex. The relationship between broccoli and cauliflower may be interpreted as forming opposite ends of a transitional gradient where improved broccoli and cauliflower group form the extremities. The glossy-leaved improved broccoli entry “Broc5” was an exception and collocated with the cluster of improved cauliflower. Chinese kale entries were readily distinguished as outliers in pairwise comparisons between Axis 3 with Axis 1 or Axis 2.

### Comparisons among botanical groups

When comparing broccoli, cauliflower, and Chinese kale, entries labeled as broccoli were the most diverse containing a total of 3543 unique SNPs, averaging 56.2 per entry (Supplementary Table ST3). Within these broccoli-specific SNPs, 2328 were exclusive to broccoli landraces (*N* = 37, average 62.9 per entry, Supplemental Figure SF2), while 195 were exclusive to improved broccoli (*N* = 26, average 7.5 per entry). The cauliflower group contained fewer unique SNPs than the broccoli group (914; *N* = 19, average 48.1 per entry). Among these cauliflower-specific SNPs, 89 were landrace-specific (*N* = 6, average 14.8) and 229 were improved specific (*N* = 13, average 17.6). Eighty-five SNPs were found to be unique to Chinese kale entries (*N* = 3, average 28.3). When comparing all landraces versus all improved entries, landraces contained on average 5.3 more unique SNPs than improved entries.

Mean adjusted fixation index, a measure of population differentiation due to genetic structure, was higher when comparing improved broccoli with improved cauliflower (F_ST_ = 0.33 ± 0.18; Supplemental Figure SF3) than when comparing all broccoli with all cauliflower entries (F_ST_ = 0.17 ± 0.12). Mean fixation index was greater when comparing landrace and improved broccoli (F_ST_ = 0.12 ± 0.08) than when comparing landrace and improved cauliflower (F_ST_ = 0.00 ± 0.10). When comparing all landraces with all improved entries, mean adjusted fixation index was relatively low (F_ST_ = 0.05 ± 0.05)

Variant-effect predictor analysis using 21,680 SNPs (F_ST_ > 0.35) identified more high-impact (HI) and moderate-impact (MI) coding variants between improved broccoli and improved cauliflower entries (HI = 66, MI = 928; Supplementary Table ST4) than when comparing all broccoli with all cauliflower (HI = 32, MI = 425; Supplementary Table ST5). More coding variants were located when comparing improved broccoli and landrace broccoli (HI = 13, MI = 167; Supplementary Table ST6) than improved cauliflower with landrace cauliflower (HI = 3, MI = 131; Supplementary Table ST7). Relatively few coding variants were located when comparing landrace and improved entries (HI = 2, MI = 16; Supplementary Table ST8).

### Linkage disequilibrium

**Fig. 4:**
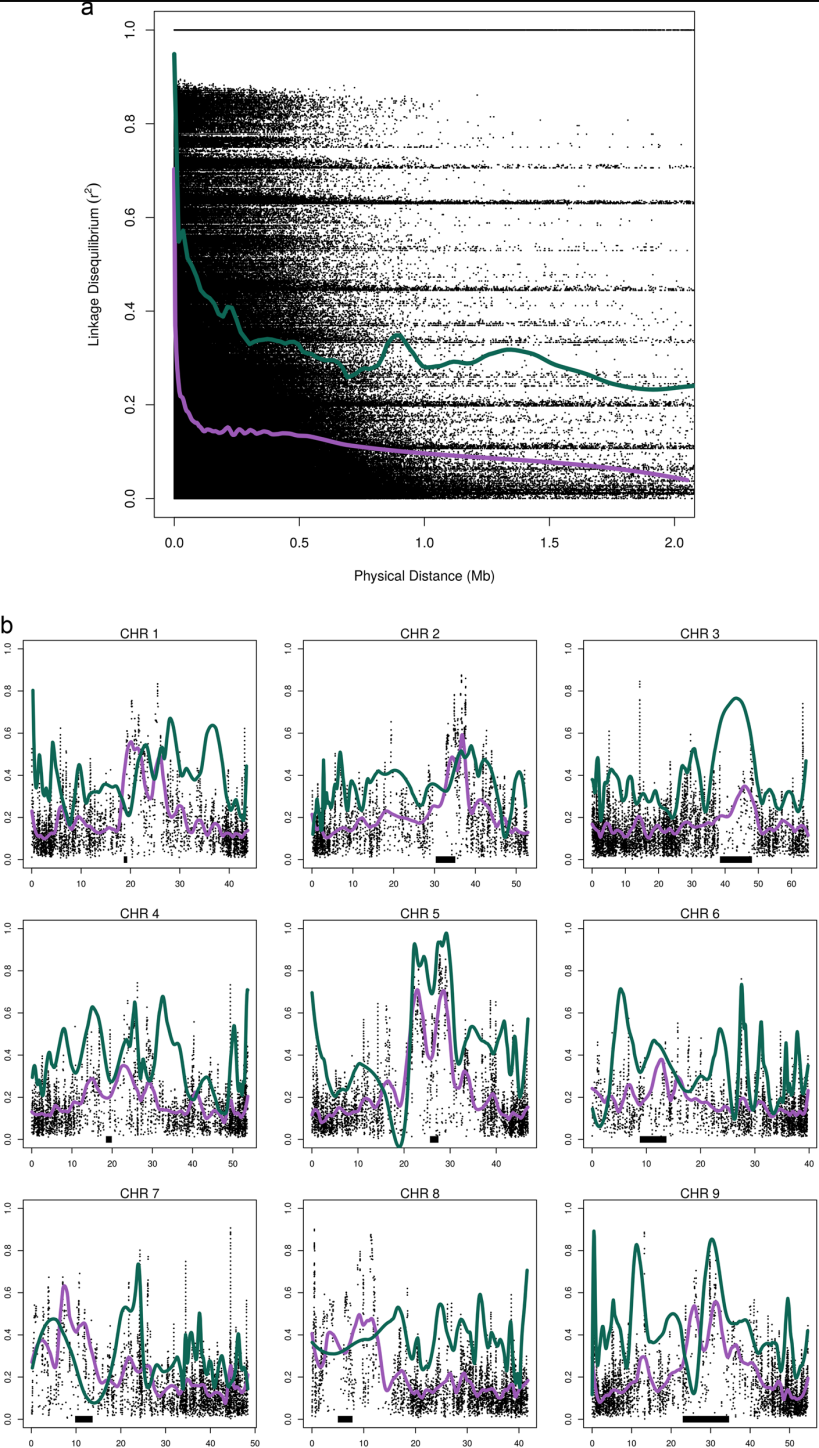
Linkage Disequilibrium Analysis. **a** Sliding window analysis (window size = 50 markers) of linkage disequilibrium (LD) decay in landrace broccoli summarized across all linkage groups and plotted against physical distance (Mb) with superimposed second-degree LOESS smoothing curves averaged over 1 Mb (green = improved broccoli; purple = landrace broccoli). **b** Genome-wide LD using all entries [*x*-axis = physical position (Mb); *y*-axis = LD (*r*^2^)] and LOESS smoothing using the same parameters plotted against LD. Putative centromere locations estimated by half-tetrad analysis^12^ are printed as horizontal black bars

We conducted linkage disequilibrium (LD) analysis in consideration of marker density required for GWAS or other mapping studies. On average, LD in landrace broccoli decayed below *r*^2^ < 0.5 by approximately 2 kb, plateaued by 200 kb and decayed to background levels below *r*^2^ < 0.1 by 900 kb (Fig. 4a), considerably faster than improved broccoli (~20, ~500, ~6450 kb respectively). Assuming a mean linkage decay of 500 kb to background levels, the *B. oleracea* v2.1 genome^12^ (~488 MB) would be divided into 976 equal haplotype blocks. On average, each haploblock would contain over 20 GBS markers using the 21,680 LD pruned markers. We observed non-uniform LD behavior within this study population most likely due to signatures of selection (e.g. domestication events) or genomic regions with reduced recombination rates such as centromeres. Several non-centromeric chromosomal regions (CHR 1, 4, 6, 7, and 9) exhibited differential LD behavior when comparing landrace broccoli with improved broccoli (Fig. 4b). GBS marker density was relatively evenly distributed genome-wide ranging from a mean of 39.6 SNPs/Mbp (CHR 2) to 51.3 SNPs/Mbp (CHR 1) (Supplementary Table ST2). Uneven GBS marker distribution (min/mean/max = 2 bp, 20.7 kbp, 463 kbp) is a likely outcome of reduced recombination frequencies of telomere and centromere regions. This level of marker density should be adequate for future GWAS; however, some variance in resolution would be expected given chromosomal location.

### Evaluating SNP Discovery

We compared SNP Discovery using the TASSEL Discovery pipeline versus the UNEAK pipeline. Discovery analysis found 203,091,048 total good barcode reads for all samples (mean = 2,194,388 ± 71,646). After merging, 1,686,226 reads remained and 65.8% were aligned to unique positions. Filtering hapmap SNPs reduced the total count to 141,317. From the UNEAK pipeline, 1,686,226 tags remained after merging, and 680,277 total tag networks were identified. A total of 115,586 reciprocal tag pairs were identified and used for SNP calling. Filtering hapmap SNPs reduced the total to 20,815 (14.7% of Discovery pipeline).

## Discussion

### Phylogenetic relationships, population structure and comparisons between botanical groups

The pool of landrace broccoli was shown to contain 61% more total polymorphic sites and 8.4 times more unique alleles per entries than hybrid broccoli. Phylogenetic and population structure analyses indicated that broccoli hybrids appear to have undergone a population bottleneck and can largely be represented by a single subpopulation within a larger and more admixed pool of broccoli germplasm. Older commercial broccoli entries such as “Green Comet” (1968), “Packman” (1983), and “Big Sur” (1980) were located relatively distantly from a concentrated cluster of more recently developed (2000 to present) broccoli hybrids^13^. Phylogenetic analysis indicated two broccoli landraces “Cavolo Broccolo Ramoso Calabrese” and “Cavolo Broccolo Verde Calbrese Precoce” appeared to be highly similar to improved broccoli. It is possible that modern broccoli was derived from lines similar to these landraces. Indeed, morphologically similar open-pollinated broccoli marketed as “Cavolo Broccolo Ramoso Calabrese” is still commercially available in Italy. The glossy-leaf broccoli entries “Broc3” and “Broc5” as well as “Zeus F1” contain membership in the red group that may reflect a lineage from a distinct genetic background. A broccoli–cauliflower cross^7^, “Green Harmony”, exhibits either a cauliflower or broccoli phenotype depending on environmental conditions and contained nearly equal membership in both the improved broccoli purple group (43.3%) and the improved cauliflower red group (47.1%). Broccoli entries were shown to contain 16.8% more average unique alleles than cauliflower entries. Chinese broccoli is effectively differentiated from a larger pool of *B. oleracea* germplasm by a third principal-coordinate axis of variation.

We conclude that an earlier or parallel domestication of broccoli compared with cauliflower is far more probable given the following observations: (a) improved cauliflower entries share more unique alleles with broccoli entries (64.3%) than cauliflower landraces share with broccoli entries (11.6%), suggesting introgression events of broccoli during the development of modern cauliflower, consistent with a broccoli-first domestication model; (b) broccoli landraces are considerably more genetically diverse in both site variants and unique alleles than other botanical groups included in this study; and (c) improved cauliflower shares a considerable population structure component with landrace broccoli.

### LD decay patterns

LD decays rapidly in landrace and improved broccoli below *r*^2^ = 0.5 (2 and 20 kb) similar to other outcrossing crop groups such as maize^14,15^ (2 kb) and grape^16^ (15 kb) but considerably slower than a collection of highly interrelated *B. oleracea* collard landraces^17^ (0.5 kb). LD in landrace broccoli plateaued roughly 2.5 times faster than improved broccoli. Given the number of high-quality SNPs and overall linkage decay, the marker saturation is suitable for future GWAS, although resolution may vary from genic to megabase levels. Moreover, this work may prove useful for identification of genes fixed during domestication that contribute to overall horticultural quality.

### Evaluating SNP Discovery

Although a high-quality reference genome proved useful for diversity analysis, 20,815 filtered markers were generated at an average density of one marker per 22.5 kb without such a resource. This result may be useful for neglected crop groups where a reference genome is unavailable or difficult to assemble.

## Materials and Methods

### Germplasm

The GBS diversity panel included 85 unique *B. oleracea* entries; of these, 63 samples were classified by passport data as broccoli (var. *italica*), consisting of 37 landraces and 26 improved entries such as F1 hybrids and doubled haploid breeding lines from public and private sources (Supplementary Table ST1). Nineteen entries were classified as cauliflower (var. *botrytis*), 6 of which were described as landraces, and 13 as improved. All cauliflower entries were either summer or fall maturing. Three entries classified as Chinese kale (var. *alboglabra*)—“TO1000” the reference genome taxa, “A12DHd Chinese Kale” an entry used in several mapping populations^8^, and “Bugh Gana” (1958) were included as a phylogenetic outgroup.

### GBS sample preparation

Seeds from each entry were germinated and grown in growth chambers under standard conditions. Young leaves were collected and subject to DNA extraction as previously described^18^. Genomic DNA of each entry was digested with *Ape*KI and used for library construction. The barcoded libraries from each entry was mixed and subjected to Illumina Next-Generation sequencing at the Cornell University Biotech Institute^11^.

### SNP production

The Tassel5 GBSv2 pipeline was run using default settings except for several modifications^19^. Minimum quality score in the GBSSeqToTagDB plugin was adjusted to 20. Chromosomes in the reference genome v2.1 (ref. ^12^) were renamed for pipeline compatibility and indexed and aligned with BWA v.0.7.8 (ref. ^20^). The BWA option samse was invoked to generate alignments in SAM format using single-end reads and randomly choosing repetitive hits. Imputation was accomplished using a K nearest neighbors^21^ approach (30 high LD sites, 10 nearest neighbors’ maximum, and a maximum of 10 Mbp between sites to search for LD). After filtering monomorphic loci and sites with more than 25% missing data, 64,323 SNPs remained. LD pruning was accomplished with PLINK (v1.90b3.46)^22^ using the indep-pairwise function with a step size of 50, a variant count of 5, and *r*^2^ threshold of 0.5, leaving 21,680 high-quality SNPs in low LD (Supplementary Table S2). Mean LD by physical position was evaluated using the “LDcorSV” package (v1.3.2) in R using *r*^2^ adjustments for population structure to generate summary statistics such as line-specific allele frequencies and botanical-group-specific alleles^23^. Pairwise genetic distances were determined using MEGA v7.0.21 (ref. ^24^).

### Diversity analysis

We partitioned our data into several datasets for subsequent analyses (Supplementary Table S3) using known passport data and an initial phenotypic screening. Principal coordinate analysis was conducted with the R package ape (v4.1)^25^. Evidence of selection^26^, allele frequency differences between genetic groups (Fixation index, F_ST_) at each locus and also across all loci were determined by the R package hierfstat (v.0.04.22)^27,28^.

To estimate the number of theoretical populations, the values of K between 1 and 10 were tested 10 times using the clustering algorithm STRUCTURE (v2.3.4) to group all varieties into the optimal population number using an admixture model assuming correlated allele frequencies^29–32^ using a burn-in of 35,000 iterations and 35,000 MCMC repetitions. Analysis output was summarized using the Cluster Markov Packager Across K algorithm (CLUMPAK)^33^ and was permuted using a Large K Greedy algorithm with random input order and 2000 repeats. CLUMPP then aligned the multiple runs of clustering to generate the best value of K using the Evanno ΔK method^34,35^. Membership coefficients for individuals and were visualized using DISTRUCT^31,36^.

An unrooted neighbor-joining tree was generated with MEGA^24^ using a maximum likelihood phylogeny reconstruction approach with 2000 bootstrap replications. All sites were used and algorithm parameters included nucleotide substitution using a general time reversible model, gamma distributed rates among sites, with four discrete gamma categories for invariant sites. The maximum likelihood heuristic was nearest-neighbor interchange with a moderate branch swap filter. The tree was visualized in FigTree^37^ using the color scheme from principal coordinate analysis.

### Comparison of GBS pipelines

#### Discovery Pipeline

To compare reference and non-reference SNP discovery, the GBS Discovery pipeline in Tassel v3.0.166 (ref. ^19^) was used with default values with several exceptions. Minimum number of tag presence was adjusted to 3 in the MergeMultipleTagCount Plugin. Maximum tag number was increased to 300,000,000 in the MergeTagsByTaxaPlugin. The proportion of taxa with at least one tag per locus was set to 0.1. Finally, the GBSHapMapFilters plugin was set to allow minimum site coverage of 0.8, maximum minor allele frequency of 1, minimum taxa coverage of 0.1, and minimum minor allele frequency of 0.01. Chromosomes in the reference genome^12^ were renamed for pipeline compatibility and indexed and aligned with BWA^20^. The BWA option samse was invoked to generate alignments in the SAM format using single-end reads and randomly choosing repetitive hits.

The non-reference based UNEAK pipeline^38^ in Tassel was also used for SNP discovery. Specifically, the genome alignment program BWA is replaced by the plugins UTagCountToTagPairPlugin and UExportTagPairPlugin. Most parameters were retained from the Discovery pipeline with several departures. The error tolerance rate in the network filter of UTagCountToTagPair plugin was set at 0.3 and the distance to pad tag pairs in the UExportTagPair plugin was adjusted to 100. The TagPairs plugin aligned sequence tags to form tag networks, where a node is comprised of a tag sequence and an edge is represented by a substitution of a single base pair between a tag pairs. A pruning algorithm was used to remove sequencing errors that may appear as low-frequency alleles. Node networks with more than two nodes were pruned to exclude multi-allelic SNP loci. The “UTagCountToTagPairPlugin” identified tag pairs using a network filter and a default error tolerance rate of 0.03. The UExportTagPairPlugin used a distance of 100 to pad tag pairs. HapMap filtering settings were adjusted to minimum site coverage = 0.8 and minimum taxa coverage = 0.1. VCFtools version (v0.1.11)^39^ was used to calculate depth and missingness from the output VCF files for both pipelines.

### Data availability

The FASTQ files of the sequencing data were deposited to NCBI SRA. SNP data are available upon request.

## Electronic supplementary material

Supplemental Figure 1: Three-dimensional plot of first three PCoA axes using 21,680 SNPs

Supplemental Figure 2: Intersection of alleles between landrace broccoli, improved broccoli, improved cauliflower, and landrace cauliflower

Supplemental Figure 3: Analyses of variant consequences for regions where FST > |0.35| between datasets

Supplemental Figure 4: Genome-wide Fst analysis between datasets plotted against physical position

Supplemental Table 1. Germplasm used within study

Supplemental Table 2. Number of high-quality LD pruned SNPs identified by Tassel 5 GBSv2 per chromosome across diversity panel compared against summary statistics from reference genome

Supplemental Table 3. Classifications of accessions used within study and number of unique 21,680 SNP dataset

Supplemental Table 4. Variants between all broccoli and all cauliflower entries

Supplemental Table 5. Variants between improved broccoli and improved cauliflower entries

Supplemental Table 6. Variants between broccoli and landrace broccoli entries

Supplemental Table 7. Variants between improved cauliflower with landrace cauliflower entries

Supplemental Table 7. Variants between landrace and improved entries

Movie. Principle component analysis
