# Supplementary material for: Genotyping-by-sequencing of Brassica oleracea vegetables reveals unique phylogenetic patterns, population structure and domestication footprints
Source: Hortic Res. 2018 Jul 1;5:38. doi: 10.1038/s41438-018-0040-3 (PMC6026498; doi:10.1038/s41438-018-0040-3)
Supplement: Supplementary file 1 — Supplemental Figure 1: Three-dimensional plot of first three PCoA axes using 21,680 SNPs [file 41438_2018_40_MOESM1_ESM.docx]

***Supplemental Figure 1:*** *Three-dimensional plot of first three PCoA axes using 21,680 SNPs. Chinese kale is represented by yellow squares, improved broccoli as green circles, landrace*

*broccoli as hollow purple circles, improved cauliflower as red triangles, landrace cauliflower as hollow orange triangles.*

***
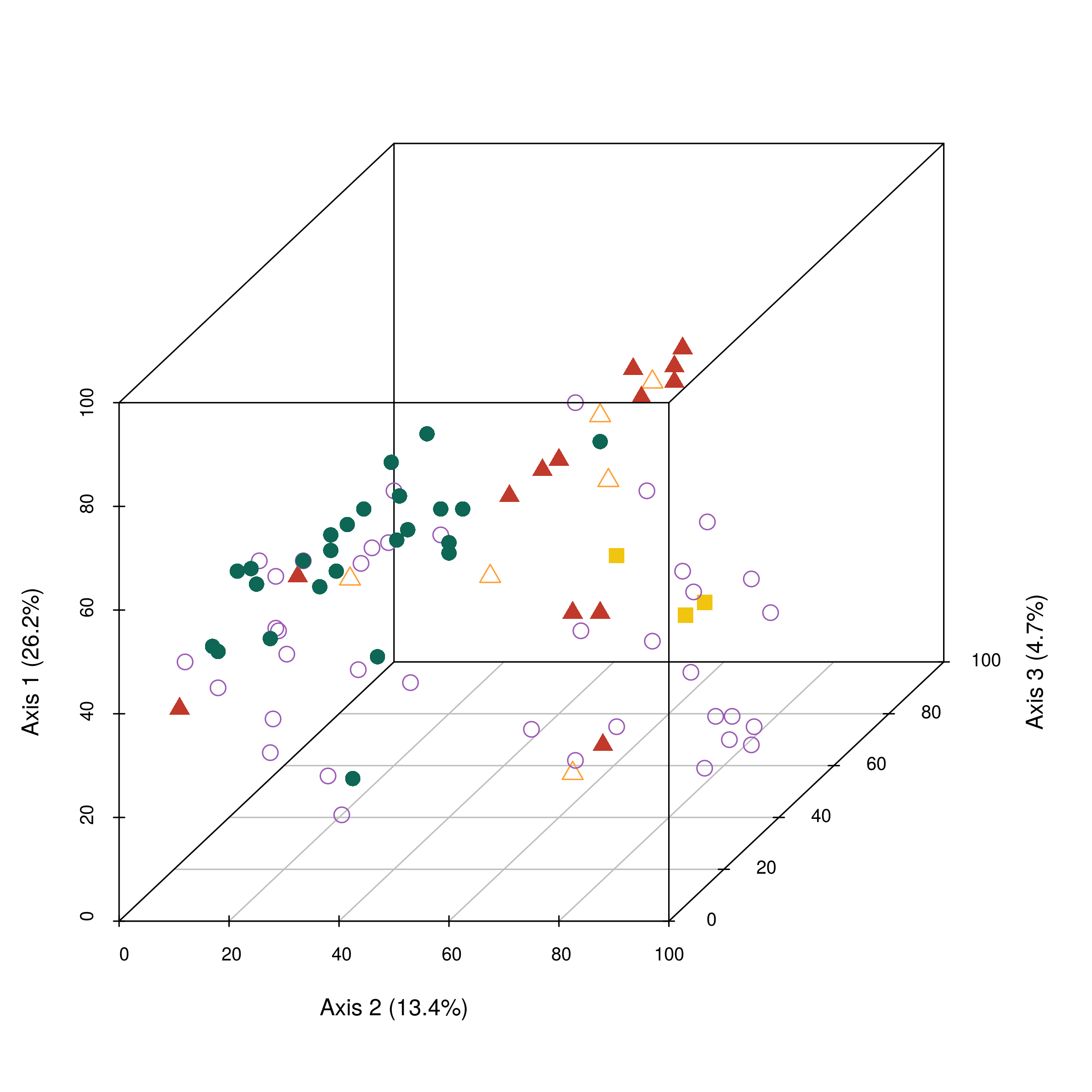
***
