# Supplementary material for: Genotyping-by-sequencing of Brassica oleracea vegetables reveals unique phylogenetic patterns, population structure and domestication footprints
Source: Hortic Res. 2018 Jul 1;5:38. doi: 10.1038/s41438-018-0040-3 (PMC6026498; doi:10.1038/s41438-018-0040-3)
Supplement: Supplementary file 2 — Supplemental Figure 2: Intersection of alleles between landrace broccoli, improved broccoli, improved cauliflower, and landrace cauliflower [file 41438_2018_40_MOESM2_ESM.docx]

***Supplemental Figure 2****: Intersection of alleles between landrace broccoli, improved broccoli, improved cauliflower, and landrace cauliflower generated by filtering 21,680 SNP group specific datasets (minimum allele frequency 0.05, minimum taxa coverage of 0.75).*

*
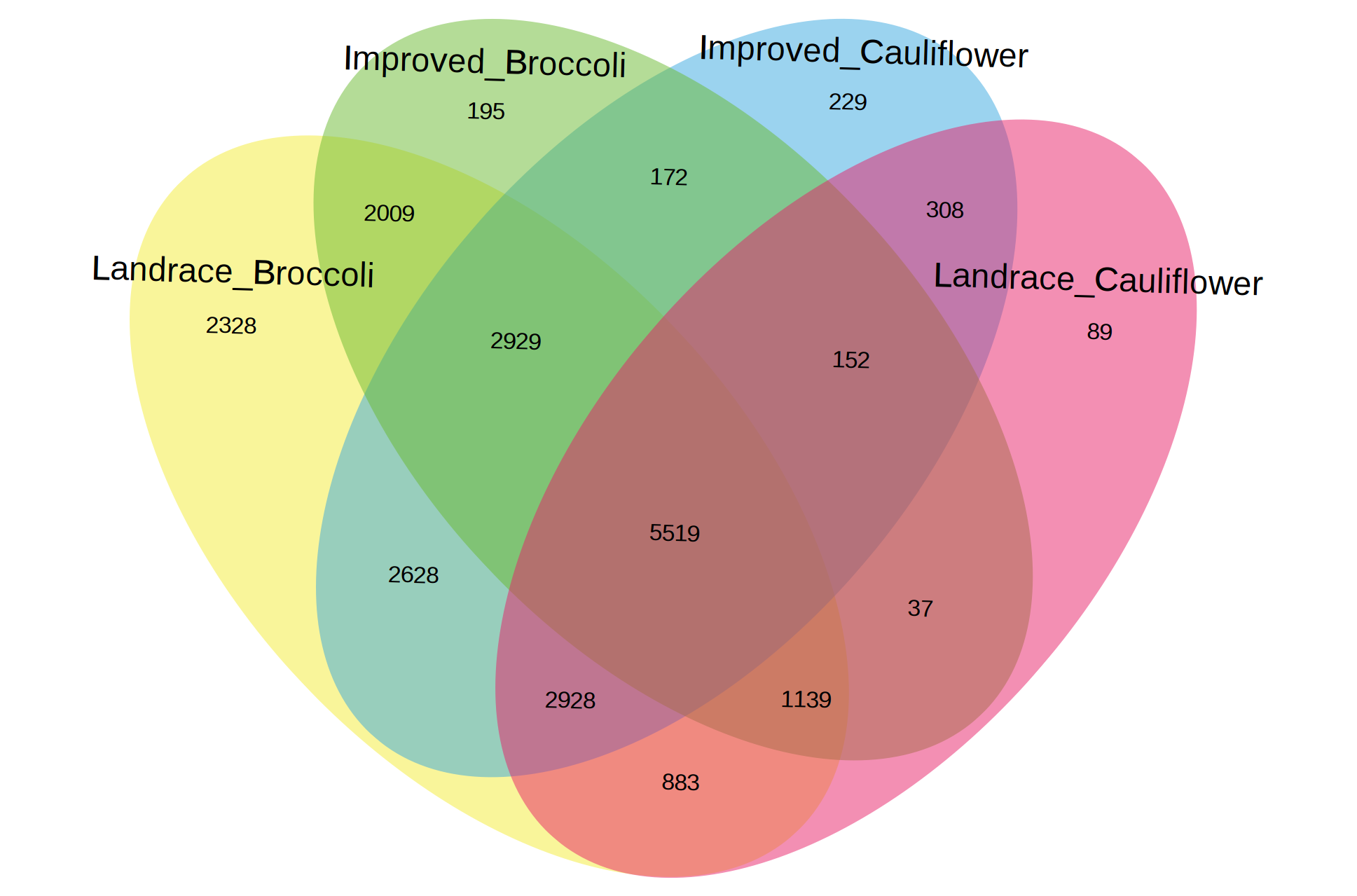
*
