# Supplementary material for: Genotyping-by-sequencing of Brassica oleracea vegetables reveals unique phylogenetic patterns, population structure and domestication footprints
Source: Hortic Res. 2018 Jul 1;5:38. doi: 10.1038/s41438-018-0040-3 (PMC6026498; doi:10.1038/s41438-018-0040-3)
Supplement: Supplementary file 3 — Supplemental Figure 3: Analyses of variant consequences for regions where FST > |0.35| between datasets [file 41438_2018_40_MOESM3_ESM.docx]

***Supplemental Figure 3****:* *Analyses of variant consequences for regions where F_ST_ > |0.35| between datasets. “Consequences (all)” applies to all loci within comparison groups, whereas “Coding consequences” applies to only loci that intersect putative coding sequences (broccoli= pooled broccoli entries, cauliflower= pooled cauliflower entries, landrace= pooled landrace entries, improved= pooled improved entries.)*

Improved Broccoli vs Landrace Broccoli (A)

Improved Broccoli vs Improved Cauliflower (B)

Broccoli vs Cauliflower (C)

Improved Cauliflower vs Landrace Cauliflower (E)

All Landraces vs All Improved (F)
