# Supplementary material for: Genotyping-by-sequencing of Brassica oleracea vegetables reveals unique phylogenetic patterns, population structure and domestication footprints
Source: Hortic Res. 2018 Jul 1;5:38. doi: 10.1038/s41438-018-0040-3 (PMC6026498; doi:10.1038/s41438-018-0040-3)
Supplement: Supplementary file 4 — Supplemental Figure 4: Genome-wide Fst analysis between datasets plotted against physical position [file 41438_2018_40_MOESM4_ESM.docx]

***Supplemental Figure 4****: Genome-wide Fst analysis between datasets plotted against physical position.*

*bIMP=Improved broccoli types, bOP= Landrace broccoli, cIMP= improved cauliflower, cOP= cauliflower landraces, broccoli= pooled broccoli entries, cauliflower= pooled cauliflower entries, landrace= pooled landrace entries, improved= pooled improved entries.*

**
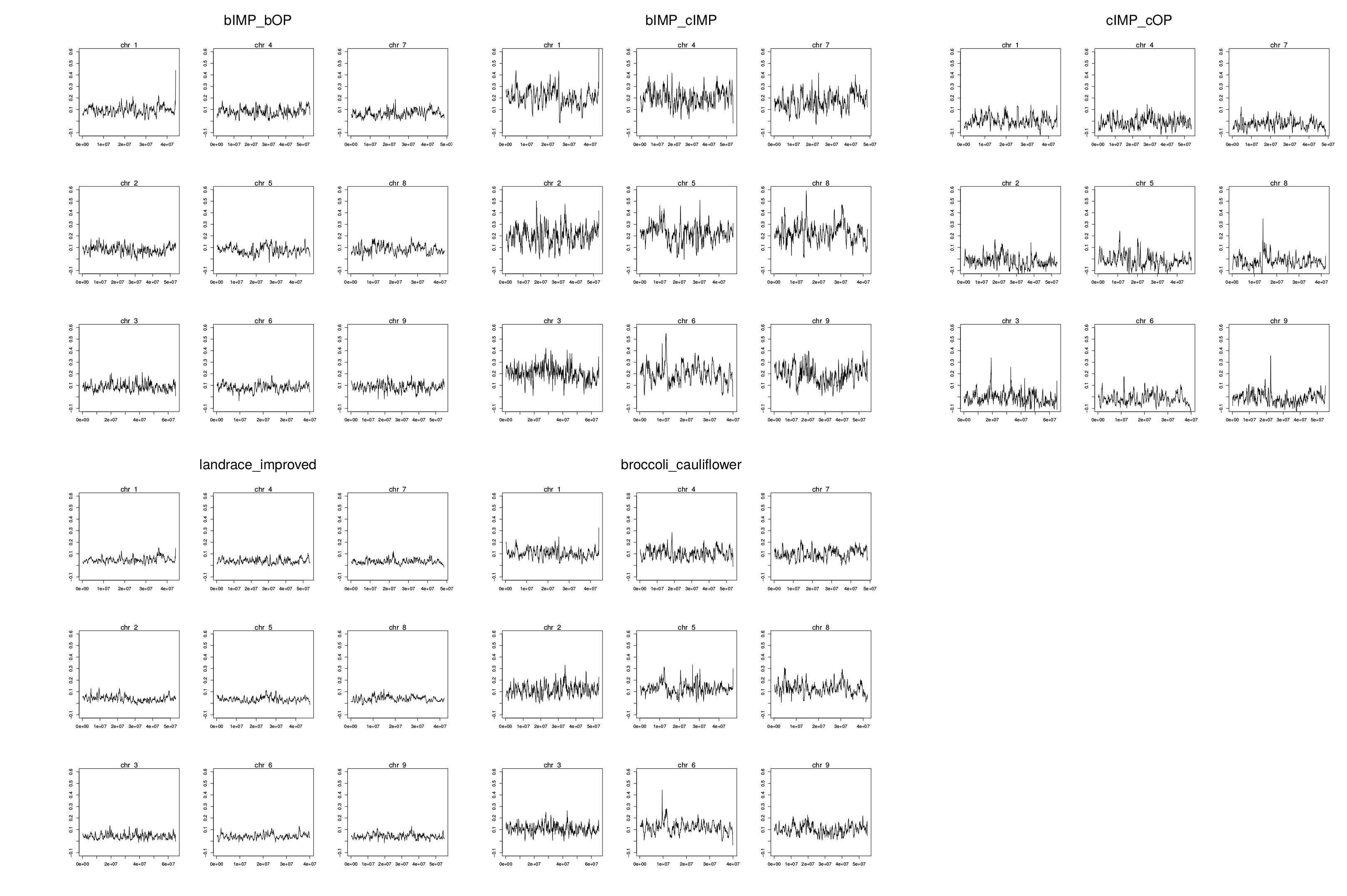
**
