# Supplementary material for: Genotyping-by-sequencing of Brassica oleracea vegetables reveals unique phylogenetic patterns, population structure and domestication footprints
Source: Hortic Res. 2018 Jul 1;5:38. doi: 10.1038/s41438-018-0040-3 (PMC6026498; doi:10.1038/s41438-018-0040-3)
Supplement: Supplementary file 5 — Supplemental Table 1. Germplasm used within study [file 41438_2018_40_MOESM5_ESM.docx]

***Supplementary Table 1.*** *Germplasm used within study.*

*Group is used for analysis of botanical group and is derived from passport information: (bIMP=Improved broccoli, bOP= Landrace broccoli, cIMP= improved cauliflower, cOP= cauliflower landraces). Notes provide additional germplasm information and accession when applicable. Breeder/Source indicates seed origin (USDA-USVL= U.S. Vegetable Laboratory, Charleston SC., HRI=Horticultural Research Institute, Wellsbourne; PRC=Plant Research Centre, Ontario; RWH= Robert W. Holley Center for Agriculture and Health, Ithaca NY; DSSC= D. Soria Seed Co., Naples Italy; MSU= Michigan State University; VT= Virginia Tech; UW= University of Wisconsin, JIC= John Innes Centre). Due to Breeder/Source consolidation, some seed companies are now merged: “*” indicates Syngenta, “†” indicates Seminis/Monsanto, “‡” indicates Limagrain Group. Year provides release date or addition to germplasm repository. Maturity indicates maturity group when applicable.*

| **Taxa** | **Group** | **Notes** | **Breeder/Source** | **Year** | **Maturity** |
| --- | --- | --- | --- | --- | --- |
| DuraPak19 F_1_ | bIMP | F_1_ Hybrid | Syngenta | 2014 | Late |
| Diplomat F_1_ | bIMP | F_1_ Hybrid | Sakata Seed Co. | 2004 | Mid-Early |
| Green Magic F_1_ | bIMP | F_1_ Hybrid | Sakata Seed Co. | 2004 | Mid-Early |
| Beaumont F_1_ | bIMP | F_1_ Hybrid | Bejo | 2003 | Late |
| Gypsy F_1_ | bIMP | F_1_ Hybrid | Sakata Seed Co. | 2002 | Early |
| Liberty F_1_ | bIMP | F_1_ Hybrid | Peto Seed Co. ^†^ | 1994 | Early |
| Major F_1_ | bIMP | F_1_ Hybrid | Royal Sluis^†^ | 1993 | Early |
| Big Sur F_1_ | bIMP | F_1_ Hybrid, PI 662786 | Asgrow Seed Co.^†^ | 1990 | Mid |
| BI10 F_1_ | bIMP | F_1_ Hybrid | Rogers Seed Co.^*^ | 1988 | Early |
| High Sierra F_1_ | bIMP | F_1_ Hybrid, G 32210 | Takii & Co. | 1988 | Late |
| Marathon F_1_ | bIMP | F_1_ Hybrid | Sakata Seed Co. | 1985 | Late |
| Packman F_1_ | bIMP | G 30778 | Peto Seed Co. ^†^ | 1983 | Early |
| Green Comet G30413 | bIMP | G 30771 | Takii & Co. | 1968 | Early |
| BC1691 F_1_ | bIMP | F_1_ Hybrid | Seminis^†^ | 2011 | Mid-Late |
| Barbados F_1_ | bIMP | F_1_ Hybrid | Ferry Morse^‡^ | 1991 | Early |
| Castle Dome F_1_ | bIMP | F_1_ Hybrid | Seminis^†^ | 2006 | Early |
| Lieutenant F_1_ | bIMP | F_1_ Hybrid | Seminis^†^ | 2011 | Early |
| Brogan F_1_ | bIMP | F_1_ Hybrid | Bejo | 1997 | Mid |
| Bay Meadows F_1_ | bIMP | F_1_ Hybrid | Syngenta | 2006 | Mid-Early |
| Zeus F_1_ | bIMP | G 30416 | Takii & Co. | < 1991 | Late |
| USVL 048 | bIMP | Inbred breeding line | USDA-USVL | 2012 | Late |
| USVL 131 | bIMP | Inbred breeding line | USDA-USVL | 2012 | Late |
| GDDH33 DH | bIMP | AGDH population, Doubled-haploid of 'Green Duke' | UK, HRI | 1993 | Early |
| Broc3 | bIMP | Glossy Leaf, PI 662578 | USA, E. Borchers, VT | < 1991 | Not Given |
| Broc5 | bIMP | Glossy Leaf, PI 662581 | Canada, D. Sampson, PRC | < 1991 | Not Given |
| Cavolfiore Violetta di Sicilia | bOP | HRI 5295 | Pugalia, Italy | 1983 | Not Given |
| GK020223 | bOP | GK020223 | UK, HRI | < 2001 | Not Given |
| BI88908 | bOP | BI88908 | UK, HRI | < 2008 | Not Given |
| Green Sprouting Early CT Strain | bOP | G 21111 | US | < 1971 | Not Given |
| Purple Sprouting Early | bOP | G 28833 | UK, HRI | < 1988 | Not Given |
| Cavolo Broccolo Bronzino di Albenga | bOP | G 28859 | Italy | < 1988 | Not Given |
| Cavolo Broccolo Ramoso Calabrese Precoce | bOP | G 28863 | Calabria, Italy | < 1988 | Not Given |
| Cavolo Broccolo Verde Calbrese | bOP | G 28865 | Calabria, Italy | < 1988 | Not Given |
| Cavolo Broccolo Frevarota | bOP | G 28872 | Italy | < 1988 | Not Given |
| Cavolo Broccolo Marzullo | bOP | G 28873 | Italy | < 1988 | Not Given |
| Broccolo di Minestra Spicata | bOP | G 28877 | Italy | < 1988 | Not Given |
| Cavolo Broccolo Natalino | bOP | G 28880 | Italy | < 1988 | Not Given |
| Cavolo Broccolo Ramoso Calabrese | bOP | G 30932 | Italy | < 1988 | Not Given |
| Cavolo Broccolo Verde Calbrese Precoce | bOP | G 30933 | Italy | < 1983 | Not Given |
| Decicco | bOP | G 32213 | Peto Seeds Inc.^†^ | < 1890 | Early |
| Indian Broccoli | bOP | PI 115881 | India | < 1936 | Early |
| Costal | bOP | PI 188561 | Brazil | < 1950 | Early |
| Ramoso | bOP | PI 441510 | Brazil | < 1978 | Early |
| Cavolo Ramoso Calabrese | bOP | PI 462206 | Italy | < 1981 | Mid |
| Broccoli di Nalale Fuglia Riccia | bOP | PI 462208 | Italy, DSSC | < 1981 | Early |
| Broccolette Neri e Cespuglio | bOP | PI 462209 | Italy | < 1981 | Late |
| Purple Sprouting Xmas | bOP | PI 662524 | UK | < 1988 | Not Given |
| Late Purple Sprouting | bOP | PI 662525 | UK | < 1988 | Not Given |
| Purple Sprouting Late | bOP | PI 662526 | UK | < 1988 | Not Given |
| Purple Sprouting Late Improved | bOP | PI 662527 | UK | < 1988 | Not Given |
| White Sprouting Early | bOP | PI 662528 | UK | < 1988 | Not Given |
| White Sprouting Improved | bOP | PI 662529 | UK | < 1988 | Not Given |
| Cavolo Ramoso Calabrese Precoce | bOP | PI 662530 | Italy | < 1988 | Not Given |
| Broccoli Neri | bOP | PI 662531 | Italy | < 1988 | Not Given |
| Broccolo Natale Pied Grande Liscio | bOP | PI 662532 | Italy | < 1988 | Not Given |
| Cavolo Broccolo Natalino di Sarno | bOP | PI 662533 | Italy | < 1988 | Not Given |
| Cavolo Broccolo di Sarno | bOP | PI 662534 | Italy | < 1988 | Not Given |
| Cavolo Cavolina Rizza | bOP | PI 662536 | Italy | < 1988 | Not Given |
| Xmas Purple Sprouting Xmas | bOP | PI 662672 | Italy | < 1990 | Not Given |
| Cavolo Broccolo Precoce G30928 | bOP | PI 662711 | Italy | < 1991 | Not Given |
| Broccoli Grande Precoce | bOP | PI 662712 | Italy | < 1984 | Not Given |
| China Broccoli G31824 | bOP | PI 662766 | China | < 1994 | Not Given |
| China Broccoli G31825 | bOP | PI 662767 | China | < 1994 | Not Given |
| Green Harmony F_1_ | cIMP | F_1_ Hybrid, Broccoli-Cauliflower, G 30769 | Taiwan, Known You Seed Co. | < 1991 | Early |
| 1227 Cauliflower | cIMP | Orange Cauliflower | USA, L. Li, RWH | < 2005 | Not Given |
| 7017 7018 Cauliflower | cIMP | Orange Cauliflower | USA, L. Li, RWH | < 2005 | Not Given |
| Absolute F_1_ | cIMP | F_1_ Hybrid, White Cauliflower | Clause Tézier Seeds | 2002 | Mid |
| ACX800 Cauliflower | cIMP | Green Cauliflower | USA, L. Li, RWH | < 2005 | Not Given |
| Cheddar F_1_ | cIMP | F_1_ Hybrid, Orange Cauliflower | Seminis^†^ | < 2005 | Early |
| EES Cauliflower | cIMP | White Cauliflower | USA, L. Li, RWH | < 2000 | Not Given |
| Fremont F_1_ | cIMP | F_1_ Hybrid, White Cauliflower | Seminis^†^ | 1995 | Early |
| Violet Queen | cIMP | G 30439 | Takii & Co. | 1991 | Mid |
| Romanesco Cauliflower | cIMP | Green Cauliflower | Bavicci Seed Co. | < 2009 | Early |
| Snow Crown F_1_ | cIMP | F_1_ Hybrid, White Cauliflower | Takii & Co. | 1975 | Early |
| Stovepipe Cauliflower | cIMP | White Cauliflower | USA, S. Honma, MSU | 1980 | Late |
| Violette Italia Cauliflower | cIMP | Purple Cauliflower | Reimer Seeds | < 2008 | Early |
| Romano | cOP | PI 231210 | Italy | < 1956 | Mid |
| Early Green Glazed | cOP | PI 234599 | South Africa | < 1956 | Early |
| Kefar Giladi | cOP | PI 291997 | Israel | < 1963 | Very Early |
| Yer Eh Fu | cOP | PI 430580 | China | < 1978 | Mid |
| Norway | cOP | PI 443022 | Norway- Torgersens Frø A/S | < 1978 | Mid |
| Violetto | cOP | PI 462222 | Italy, DSSC | < 1981 | Early |
| A12DHd Chinese Kale | Chinese kale | Doubled-Haploid | UK, D. Keith, JIC | < 1996 | Very Early |
| TO1000 | Chinese kale | Doubled-Haploid | USA, P. Williams, UW | 1986 | Very Early |
| Bugh Gana | Chinese kale | PI 249556 | Thailand | < 1958 | Very Early |
