# Supplementary material for: Genotyping-by-sequencing of Brassica oleracea vegetables reveals unique phylogenetic patterns, population structure and domestication footprints
Source: Hortic Res. 2018 Jul 1;5:38. doi: 10.1038/s41438-018-0040-3 (PMC6026498; doi:10.1038/s41438-018-0040-3)
Supplement: Supplementary file 6 — Supplemental Table 2. Number of high-quality LD pruned SNPs identified by Tassel 5 GBSv2 per chromosome across diversity panel compared against summary statistics from reference genome [file 41438_2018_40_MOESM6_ESM.docx]

***Supplemental Table 2.*** *Number of high-quality LD pruned SNPs identified by Tassel 5 GBSv2 per chromosome across diversity panel compared against summary statistics from reference genome.*

| Chromosome | SNPs | Chr Size (Mbp) | Density (SNP/Mbp) |
| --- | --- | --- | --- |
| 1 | 2283 | 44.5 | 51.3 |
| 2 | 2132 | 53.8 | 39.6 |
| 3 | 3657 | 65.8 | 55.6 |
| 4 | 2540 | 55.3 | 45.9 |
| 5 | 2293 | 48.4 | 47.4 |
| 6 | 1889 | 40.4 | 46.8 |
| 7 | 2256 | 49.6 | 45.5 |
| 8 | 1958 | 43.4 | 45.1 |
| 9 | 2672 | 56.1 | 47.6 |
| Total | 21680 | 457.3 | 47.4 |
