# Supplementary material for: Genotyping-by-sequencing of Brassica oleracea vegetables reveals unique phylogenetic patterns, population structure and domestication footprints
Source: Hortic Res. 2018 Jul 1;5:38. doi: 10.1038/s41438-018-0040-3 (PMC6026498; doi:10.1038/s41438-018-0040-3)
Supplement: Supplementary file 7 — Supplemental Table 3. Classifications of accessions used within study and number of unique 21,680 SNP dataset [file 41438_2018_40_MOESM7_ESM.docx]

***Supplemental Table 3.****Classifications of accessions used within study and number of unique 21,680 SNP dataset.*

*Comparisons between****crop groups in bold****, within crop group underlined (bIMP=Improved broccoli types, bOP= Landrace broccoli, cIMP= improved cauliflower, cOP= cauliflower landraces, Albog= Chinese kale). Landrace and improved comparisons are marked with an apostrophe.*

| **Dataset** | **Entries** | **N** | **LD Pruned SNPs** | **Unique SNPs** | **Unique/ N** |
| --- | --- | --- | --- | --- | --- |
| All | bIMP, bOP, cIMP, cOP, Albog | 85 | 21680 | NA | NA |
| **Broccoli** | bIMP, bOP | 63 | **20200** | **3543** | **56.2** |
|  | bIMP | 26 | 12092 | 195 | 7.5 |
|  | bOP | 37 | 19478 | 2328 | 62.9 |
| **Cauliflower** | cIMP, cOP | 19 | **17038** | **914** | **48.1** |
|  | cIMP | 13 | 14805 | 229 | 17.6 |
|  | cOP | 6 | 10959 | 89 | 14.8 |
| **Chinese Kale** | Albog | 3 | **5141** | **85** | **28.3** |
| **Pooled Landrace*** | bOP, cOP | 38 | **20556** | 4121 | 108.4 |
| **Pooled Improved*** | bIMP, cIMP | 43 | **17332** | 887 | 20.6 |
