# Supplementary figures and images for: Genotyping-by-sequencing of Brassica oleracea vegetables reveals unique phylogenetic patterns, population structure and domestication footprints
Source: Hortic Res. 2018 Jul 1;5:38. doi: 10.1038/s41438-018-0040-3 (PMC6026498; doi:10.1038/s41438-018-0040-3)

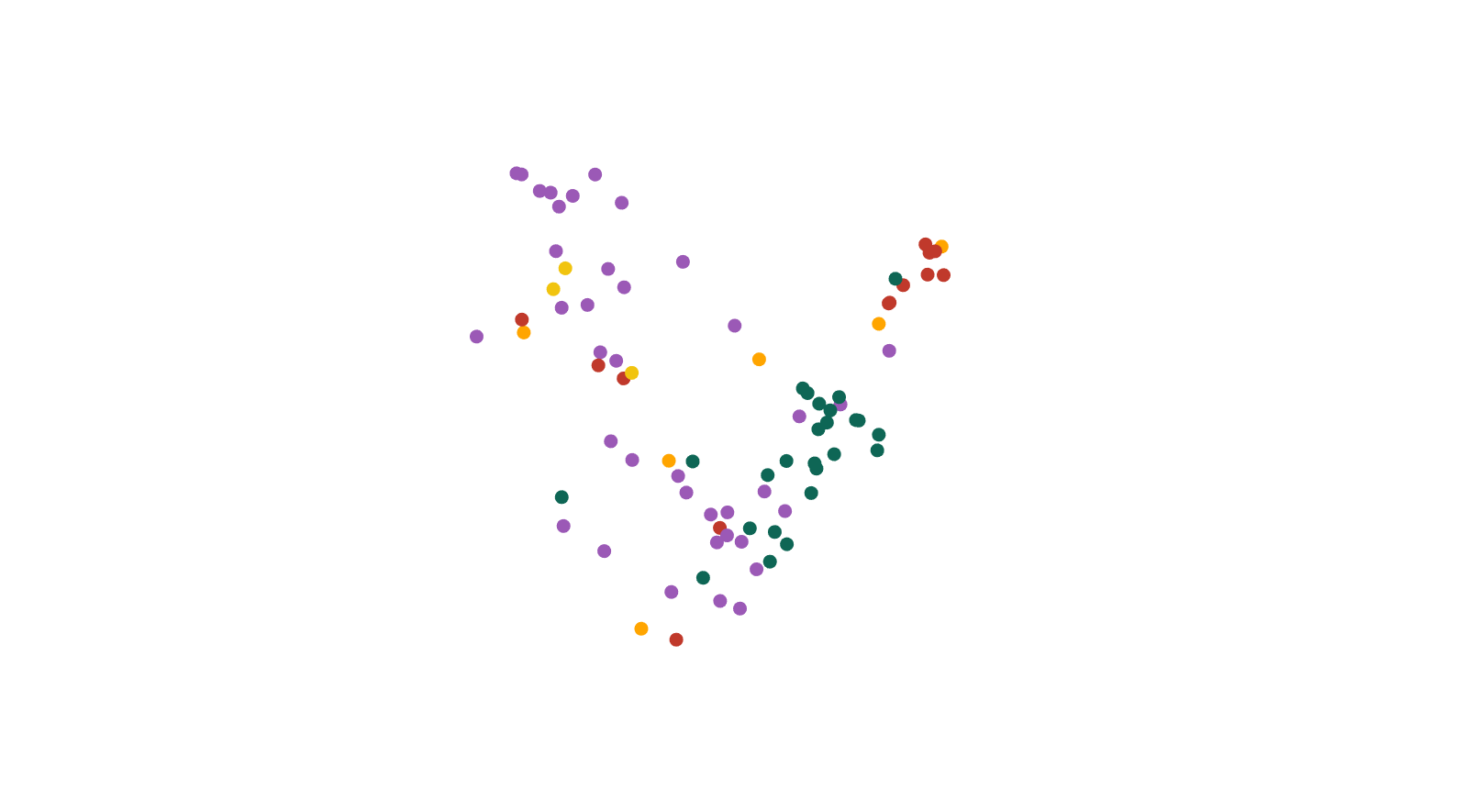

Supplement: Supplementary file 13 — Movie. Principle component analysis [file 41438_2018_40_MOESM13_ESM.gif]
